# Etiological and Clinical Characteristics of HLA-B27-associated Uveitis in a Tertiary Referral Center

**DOI:** 10.4274/tjo.galenos.2018.53896

**Published:** 2019-02-28

**Authors:** Merve İnanç, Mert Şimşek, Müge Pınar Çakar Özdal

**Affiliations:** 1 Erciş State Hospital, Ophthalmology Clinic, Van, Turkey; 2 Ulucanlar Eye Training and Research Hospital, Ophthalmology Clinic, Ankara, Turkey

**Keywords:** HLA-B27, uveitis, ankylosing spondylitis, demography, etiology

## Abstract

**Objectives:**

To investigate the demographic, etiologic, and clinical features of HLA-B27-associated uveitis.

**Materials and Methods:**

The clinical records of 91 patients diagnosed with HLA-B27-associated uveitis at the Ulucanlar Eye Training and Research Hospital between the years of 2005 and 2016 were reviewed. Each patient’s presenting complaints, best-corrected visual acuities in first and last visits, biomicroscopic and fundoscopic examination findings, frequency and seasonal distribution of attacks, and demographic data such as age and sex were noted. Therapeutic approaches, duration of follow-up, and complications were analyzed.

**Results:**

A total of 91 patients (179 eyes) aged 19-82 years (mean age 46.52±13.06 years) were included. Forty-three patients (47.3%) were female and 48 (52.7%) were male. Bilateral involvement was observed in 44 (48.4%) and unilateral involvement was observed in 47 (51.6%) patients. The most frequent complaint was redness (67%), followed by decreased and/or blurred vision (50.5%). The mean follow-up time was 38.2 months (range, 1-245 months). Anterior uveitis was most common anatomical subtype, seen in 86 (94.5%) of the patients. Mean number of attacks was 1.93±1.45 per patient-year and a significantly higher number of uveitis attacks (47%) occurred in winter. Twenty-four patients (26.3%) were diagnosed with ankylosing spondylitis. Fibrinous uveitis was detected in 36 patients (39.5%). Posterior synechia developed in 41 (22.9%) and hypopyon developed in 7 (3.9%) eyes. The most common complications were cataract (n=12, 6.7%) and ocular hypertension (n=15, 8.3%).

**Conclusion:**

Ninety-one (6.3%) of the 1422 patients followed in our uvea clinic were diagnosed with HLA-B27-associated uveitis. HLA-B27-associated uveitis is characterized by acute, recurring sudden-onset iridocyclitis with a moderate to severe amount of fibrin and cells in the anterior chamber, and is easily treatable. Visual prognosis is good despite the complications.

## Introduction

Uveitis, which is defined as intraocular inflammation, predominantly affects the working-age population (20 to 50 years old) and leads to substantial individual and socio-economic burdens.^1^ Anatomic location of inflammation, disease course, and the presence of comorbid infectious or systemic disease should be considered when evaluating a patient with uveitis.^2^

The most common form is anterior uveitis and the most common subtype is acute anterior uveitis (AAU).^3^ HLA-B27-associated uveitis is the most common cause of anterior uveitis overall and of anterior uveitis with hypopyon in particular.^4,5,6^ The diagnosis of HLA-B27-associated AAU is based on clinical findings and positive HLA-B27 antigen test after ruling out other infectious or inflammatory diseases. In spite of ongoing research, the pathogenesis of HLA-B27-associated uveitis is not fully understood. In addition, patients with HLA-B27-associated uveitis vary in terms of their clinical features, response to treatment, and systemic comorbidities.^7^

The aim of this study was to identify the clinical spectrum and related systemic diseases in HLA-B27-associated uveitis by investigating the etiological and clinical features of patients who presented to a tertiary eye care center and were diagnosed with HLA-B27-associated uveitis.

## Materials and Methods

The clinical records of 91 patients who were diagnosed with HLA-B27-associated uveitis and followed for at least 6 months in the Ulucanlar Eye Hospital between the years 2005 and 2016 were evaluated retrospectively. The study protocol was approved by the ethics committee and the study was performed according to the Declaration of Helsinki. Written informed consent was obtained from each participant.

In all cases, the presence of Behçet’s disease, viral anterior uveitis demonstrated with clinical findings and laboratory tests when necessary, syphilis (which can mimic any clinical condition), and sarcoidosis and tuberculosis etiologies in clinically suspicious cases were ruled out. All patients were questioned about backache and morning stiffness, and rheumatology consultation was requested to evaluate for the potential comorbidities ankylosing spondylitis (AS) and other spondyloarthropathies, as well as to test for HLA-B27 positivity. Seven patients who were followed for less than 6 months were excluded.

The patients were analyzed in terms of demographic data such as sex and age at uveitis onset, presenting complaints, best corrected visual acuity (BCVA) at initial and final visits as measured by Snellen scale, intraocular pressure (IOP) measured using a noncontact tonometer, and detailed ophthalmological examination findings including slit-lamp and fundus examination findings. 

Ocular involvement was classified as recurrent in the same eye (unilateral), recurrent in both eyes at different times (bilateral alternating), or recurrent in both eyes at the same time (bilateral simultaneous). During acute episodes, the IOP values in both eyes were compared. Patients with posterior synechia, hypopyon, and/or fibrinous reaction in the anterior chamber were recorded separately. The frequency and seasonal distribution of uveitis attacks were noted. Follow-up time, systemic comorbidities, therapeutic approaches, complications, and surgical procedures were also analyzed.

IOP over 21 mmHg in two examinations was defined as ocular hypertension in the absence of detectable visual field defect and as secondary glaucoma when accompanied by visual field loss. Complicated cataract induced by uveitis was defined as posterior subcapsular opacity that developed subsequently in the lens.

### Statistical Analysis

The data were analyzed using Statistical Package for the Social Sciences version 22.0 software (SPSS Inc., Chicago, IL, USA). Mean values, percentages, and chi-square test were used for statistical analyses. P value less than 0.05 was considered significant.

## Results

Ninety-one of 98 patients aged 19-82 years (mean 46.52±13.06 years) were included in the study. Demographic and clinical features of the patients are presented in Table 1. Forty-three (47.3%) of the patients were women and 48 (52.7%) were men. Patients diagnosed with HLA-B27-associated uveitis comprised 6.3% (91/1422) of the patients under follow-up in our uvea unit.

Presenting symptoms of the patients are shown in Table 2. The most common presenting symptom was redness (67%), followed by reduced visual acuity (50.5%). Mean follow-up time was 38.2 (6-245) months. Forty-four (48.4%) of the patients had bilateral and 47 (51.6%) had unilateral involvement. Fourteen (31.8%) of the 44 patients with bilateral involvement had simultaneous episodes, while the remaining (68.2%) had bilateral alternating involvement. In terms of anatomic location, anterior uveitis was most common, detected in 86 (94.5%) of the patients. Of the remaining 5 patients, 3 had intermediate uveitis and 2 had panuveitis.

**Figure 1:**
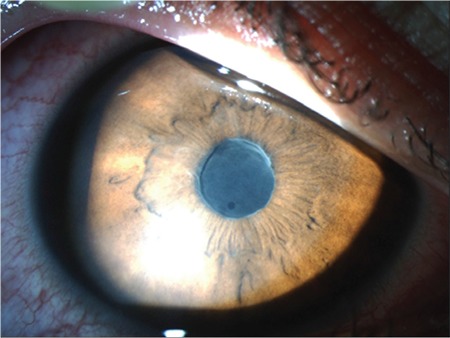
Anterior segment photograph of a patient with HLA-B27-associated uveitis showing broad-based posterior synechia

**Figure 2:**
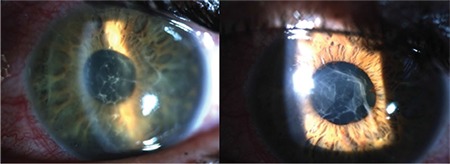
Anterior segment photograph of a patient with HLA-B27-associated uveitis showing fibrinous uveitis (left) and regression of the fibrinous reaction after 3 days of topical therapy (right)

**Figure 3:**
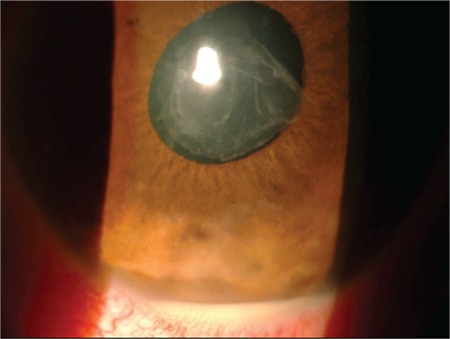
Anterior segment photograph of a patient with HLA-B27-associated uveitis showing hypopyon

Of the 135 eyes with acute uveitis episodes, there was a significant difference between mean initial BCVA (0.2±0.37) and mean final BCVA (0.11±0.25) converted to LogMAR (logarithm of the minimum angle of resolution) (p<0.0001). Clinical findings included posterior synechia in 41 patients (22.9%) (Figure 1), presence of fibrin during acute episodes in 36 patients (39.5%), (Figure 2) and hypopyon in 7 patients (3.9%) (Figure 3). Posterior synechia was present in 35 eyes (85.4%) at presentation and developed during follow-up in 6 eyes (14.6%). During acute episodes, IOP in the uveitic eye was ≥5 mmHg lower than in the nonuveitic eye in 17 (18.6%) patients and ≥5 mmHg higher in 15 (16.4%) patients. In terms of posterior segment findings, macular edema was observed in 1 patient.

The mean number of acute episodes per year was 1.93±1.45 and all patients had at least 1 attack during follow-up. Evaluation of the seasonal distribution of attacks showed that they occurred most often in the winter months (47%), followed by summer (27.3%), spring (16.2%), and autumn (9.5%). 

With regard to medical treatment, acute episodes were treated in all patients with a topical steroid and cycloplegic agent at appropriate doses based on the severity of inflammation. In addition, 23 eyes (12.8%) received subconjunctival injection for severe anterior segment inflammation, 1 eye (0.6%) received sub-Tenon’s injection for macular edema, and 9 eyes (5.0%) were treated with topical antiglaucomatous therapy due to ocular hypertension. Twenty-nine patients (31.8%) also received oral indomethacin 2 or 3 times daily depending on severity of inflammation. A total of 6 (6.6%) patients were given oral steroid therapy, 5 (5.5%) for severe ocular inflammation and 1 (1.1%) for macular edema. All patients diagnosed with AS were taking sulfasalazine (Salazopyrin). The rheumatology department prescribed oral steroid therapy to 2 patients (2.2%), methotrexate to 3 (3.3%), cyclosporine to 1 (1.1%), and infliximab to 1 patient (1.1%).

Systemic comorbidities included AS in 24 patients (26.3%), undifferentiated spondyloarthropathy in 8 patients, rheumatoid arthritis in 2, psoriasis in 1, and Crohn’s disease in 1 patient. Fibrinous reaction was not associated with presence of AS (p=0.291) (Table 3). Prevalence rates of ocular complications are presented in Table 4. The most common complications during follow-up were complicated cataract (n=12, 6.7%) and ocular hypertension (n=15, 8.3%). Eight eyes (4.4%) underwent phacoemulsification and intraocular lens implantation, while 1 (0.56%) underwent trabeculectomy due to secondary glaucoma.

## Discussion

The relationship between HLA-B27 positivity and inflammatory diseases such as uveitis, AS, reactive arthritis, ulcerative colitis, and psoriasis was first identified in 1973.^8,9,10,11^ There are substantial global differences in the prevalence of HLA-B27 antigen. These differences also explain observations of different global patterns of uveitis. In their publication on the demographic and clinical properties of uveitis in Turkey, Yalçındağ et al.^12^ reported the prevalence of HLA-B27 antigen in the Turkish population as 6.8% and that HLA-B27-associated uveitis accounted for 3.9% of all cases of uveitis. In their epidemiological study of uveitis, Özdal et al.^13^ determined that HLA-B27-associated uveitis comprised 4.6% of uveitis cases. The proportion of HLA-B27-associated uveitis in the present study was 6.3% of all uveitis cases. The lower rates reported in previous studies may be due to the exclusion of uveitis associated with AS and other spondyloarthropathies. 

HLA-B27-associated uveitis is characterized by symptomatic unilateral anterior uveitis with sudden onset and limited duration. Our study also showed that patients with HLA-B27-associated uveitis were symptomatic at time of presentation, that anterior uveitis was the most common type based on anatomic location, and most patients exhibited unilateral involvement. The majority of studies in the literature concerning sex distribution have reported male predominance. In the Turkish population, Kazokoglu et al.^13^reported a slight female predominance, whereas Yalçındağ et al.^12^ and Tuncer et al.^14^ observed male predominance, consistent with our study. 

In active periods of HLA-B27-associated uveitis, IOP is expected to be lower due to ciliary body inflammation and reduced aqueous production. van der Veer et al.^15^ reported 5 patients who developed hypotony and serous retinal detachment secondary to HLA-B27-associated anterior uveitis, while Roe et al.^16^ reported 1 case with hypotony maculopathy. However, IOP may be high due to trabeculitis, accumulation of inflammatory cells and waste in the trabecular network, and/or steroid use. In the present study, IOP was lower in the eye with acute uveitis in 18.6% and higher in 16.4% of the patients.

In the literature related to posterior segment involvement in HLA-B27-associated uveitis, Rothova et al.^17^ reported detecting no posterior segment involvement in 153 patients with seronegative spondyloarthropathies and/or HLA-B27-associated uveitis, and Mapstone and Woodrow^18^ reported involvement in only 2 (3.9%) of 51 patients. In a cohort study of 166 patients with HLA-B27-associated uveitis, Rodriguez et al.^19^ described 29 patients (17.4%) with posterior segment inflammatory findings. They attributed this high rate to the fact as a tertiary health center, referred cases were particularly difficult and complex. In our department, which is in one of the largest tertiary health centers in Turkey, panuveitis accompanied by vitreous haze and retinal vasculitis was observed in 2 patients and macular edema with severe anterior segment reaction was detected in only 1 patient.

A study examining the Turkish patient population in terms of the clinical features of HLA-B27 positive and negative AAU patients showed that 7% of HLA-B27 positive AAU cases had concurrent bilateral AAU, whereas this proportion was higher in our study (31.8%).^14^The prevalence of AS in HLA-B27-associated AAU was shown to be 22-39% in several studies.^20,21,22^ Tuncer et al.^14^ determined this rate to be 43% in their study on the Turkish population, and we found this rate to be 26% in our series. Half of HLA-B27 positive AAU patients can develop uveitis before the onset of spondylitis, with an average of 3 years between uveitis and spondylitis onset.^22,23,24^ Our findings also support the importance of taking a detailed history and conducting a thorough investigation for potential systemic comorbidities in all patients with HLA-B27-associated uveitis. There are publications showing that HLA-B27 positive patients with systemic disease have a higher probability of recurrent uveitis episodes than those without systemic symptoms.^20,25^ However, Gehlen et al.^26^ reported that uveitic activity may not always be related to the activity of systemic HLA-B27-associated disease. In the present study, we observed no statistically significant difference in attack frequency between patients with and without systemic disease (p>0.05).

Inflammation in HLA-B27-associated uveitis generally responds well to topical therapy. However, patients sometimes present with hypopyon, fibrinous reaction in the anterior chamber, and pupillary seclusion; response to topical therapy is insufficient in these cases. HLA-B27 antigen positivity was detected by Power et al.^20^ in 14.1% and by D’Alessandro et al.^27^ in 14.5% of anterior uveitis patients presenting with hypopyon. In our series, hypopyon was observed in 3.4% of patients. The formation of fibrinous membrane in the absence of granulomatous precipitate was put forth as a classic presentation of HLA-B27-associated anterior uveitis.^18^ However, Huhtinen and Karma^28^ reported that fibrinous reaction was not more frequent in HLA-B27 positive unilateral AAU cases (43%) than in idiopathic HLA-B27 negative unilateral AAU cases (55%). Tuncer et al.^16^ observed fibrinous reaction in 39% of HLA-B27 positive uveitis patients in the Turkish population, and this rate was 39.5% in our series.

There are studies in the literature analyzing changes in the seasonal distribution of AAU attacks.^30,31,32^ Two of these studies evaluated the effect of HLA-B27 positivity on the seasonal distribution of AAU attacks, but their results were conflicting.^30,31^ Chung et al.^31^ evaluated only HLA-B27 positive AAU cases and found that recurrence was more likely in December through March (winter). In a population-based study investigating monthly variations in AAU cases, a statistically significant increase in the number of AAU attacks was detected in December in two consecutive years. Comparison of HLA-B27 positive and negative AAU cases revealed no statistically significant difference in monthly distribution between the two groups, but attacks tended to recur more in late winter in HLA-B27 positive patients.^32^ We also observed in the present study that attacks were more common in the winter months. This difference in the seasonal distribution of AAU attacks suggests that weather conditions may be a factor.

## Conclusion

In conclusion, our study suggests that HLA-B27-associated uveitis is characterized by anterior segment inflammation that has limited duration and shows unilateral or alternating bilateral involvement, and that uveitis may coexist with systemic diseases, particularly AS. Despite the development of complications, visual prognosis is good with appropriate treatment to control inflammation.

## Figures and Tables

**Table 1:**
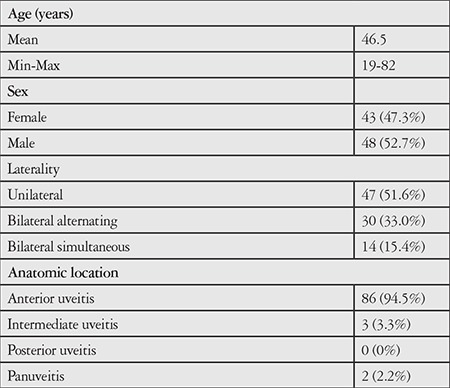
Demographic and clinical characteristics of the patients

**Table 2:**
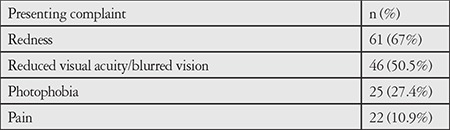
Patients’ symptoms at presentation

**Table 3:**
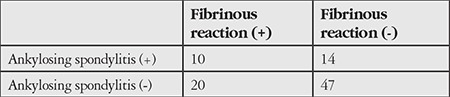
Relationship between ankylosing spondylitis and fibrinous reaction

**Table 4:**
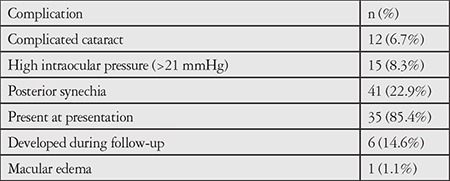
Structural ocular complications in HLA-B27- associated uveitis
